# HAT-PCR Enables Sensitive Quantification of Minimal Residual Disease in Chronic Lymphocytic Leukemia and Myeloma

**DOI:** 10.3390/ijms26167720

**Published:** 2025-08-10

**Authors:** Elizabeth Hughes, Sue Latham, Bryone Kuss, Scott Grist, Rachel Hall, Tiffany Khong, Malgorzata Gorniak, Andrew Spencer, Constantine Tam, Stephen Mulligan, Sheree Bailey, Mary Sartor, Dennis Carney, Gavin Cull, David Gottlieb, Alexander Morley

**Affiliations:** 1 School of Medicine, Flinders University, Adelaide 5042, Australia; elizabeth.hughes@flinders.edu.au (E.H.); suelatham@internode.on.net (S.L.); bryone.kuss@flinders.edu.au (B.K.); sheree.bailey@unisa.edu.au (S.B.); 2 Genomix, Adelaide 5042, Australia; scott.grist@gnomix.com.au (S.G.); rachel.hall@gnomix.com.au (R.H.); 3 Alfred Hospital, Melbourne 3181, Australia; m.gorniak@alfred.org.au (M.G.); andrew.spencer@monash.edu (A.S.); constantine.tam@monash.edu (C.T.); 4 School of Medicine, University of Sydney, Sydney 2006, Australia; stephen.mulligan@sydney.edu.au (S.M.); mary.sartor@outlook.com (M.S.); david.gottlieb@sydney.edu.au (D.G.); 5 Peter MacCallum Clinic, Melbourne 3000, Australia; dacarney@unimelb.edu.au; 6 Sir Charles Gairdner Hospital, Perth 6009, Australia; gavin.cull@health.wa.gov.au

**Keywords:** PCR, flow cytometry, acute lymphoblastic leukemia, chronic lymphocytic leukemia, myeloma, minimal residual disease, measurable residual disease, next generation sequencing, digital PCR

## Abstract

The role of HAT-PCR (**H**igh **A**/**T** or **H**igh **A**nnealing **T**emperature–PCR) in the quantification of minimal residual disease (MRD) was investigated in chronic lymphocytic leukemia (CLL) and myeloma. The IGH gene sequence was determined by next-generation sequencing (NGS), either by the Lymphotrack kit or by preparing libraries using an in-house two-round PCR protocol which enabled successful sequencing in 37/37 CLL marrow samples and 34/35 myeloma marrow samples. MRD was quantified by HAT-PCR in 125 CLL marrow or blood samples from 36 patients, with 2 results being less than 10^−6^ and in 63 myeloma marrow samples from 35 patients, with 10 results being less than 10^−6^. Measurement of MRD in 113 pairs of CLL samples and 51 pairs of myeloma samples showed that HAT-PCR was significantly more sensitive than flow. Compared to marrow MRD, blood MRD was relatively high in CLL but very low or undetectable in myeloma. Flow-positive HAT-PCR negative samples were not seen in myeloma, although the literature review suggested that flow-positive NGS-negative myeloma samples are sometimes observed. The ability of HAT-PCR to quantify down to and below 10^−6^ and the practical advantages of PCR suggest that HAT-PCR could be used widely for the quantification of MRD in lymphoid malignancy.

## 1. Introduction

The quantification of minimal residual disease (MRD) is widely used in malignant hematological disorders. In acute lymphoblastic leukemia (ALL), MRD is used both for making decisions on treatment for individual patients and for research in monitoring response in clinical trials. In chronic lymphocytic leukemia (CLL) and myeloma, MRD is routinely used in clinical trials, but its use for the management of individual patients is still uncertain.

Since the original report [1], PCR using patient-specific primers directed to the rearranged immunoglobulin or T cell receptor has been used to quantify MRD up to the present time, most notably by the EuroMRD consortium [2,3,4]. The original report quantified what is now termed digital PCR (dPCR) [5,6], but real-time quantitative PCR (RT-qPCR) [7] rapidly became the method of choice owing to its greater convenience, although dPCR is again starting to be used following the development of new instrumentation [8].

As it is usually performed, RT-qPCR can reliably quantify MRD down to a level of 10^−4^–10^−5^ and digital PCR can probably quantify to a slightly lower level. Neither method has been able to quantify MRD below a level of 10^−5^, and there is even some doubt for results in the range of 10^−4^–10^−5^, as a proportion of them appear to be false positives [9,10]. This limited sensitivity of PCR has been a stimulus to the measurement of MRD being performed by more sensitive techniques such as flow cytometry [11,12], which can usually quantify down to 10^−5^, and next-generation sequencing (NGS) [13,14,15], which can usually quantify down to 10^−6^.

HAT-PCR (**H**igh **A**/**T** or **H**igh **A**nnealing **T**emperature–PCR) is a variant of PCR in which the primers and protocol are designed to produce increased specificity [16]. The false-positive results sometimes seen with conventional PCR are prevented or minimized, as is the promiscuous binding of the PCR primers to sequences in genomic DNA, which, particularly when the MRD level is low, can result in the silent amplification of non-specific material to such an extent that the PCR becomes inhibited [17]. HAT-PCR quantified down to 10^−6^ in the laboratory and, when investigated in adult ALL, the measurement of MRD in blood by HAT-PCR was non-inferior to the measurement of MRD in marrow by conventional PCR, both in terms of detecting disease and in predicting clinical outcome [16]. As the level of MRD in blood is approximately one log lower than that in marrow, this result suggested that HAT-PCR is more sensitive than conventional PCR.

In view of the suggested sensitivity of HAT-PCR and the practical advantages of cost, timeliness, and availability provided by PCR, we have investigated the potential wider role of HAT-PCR in the measurement of MRD by quantifying MRD in samples from patients with CLL and myeloma in order to obtain further data on the sensitivity of the method, and by directly comparing the sensitivity of HAT-PCR to that of flow cytometry, which is a standard method used for the quantification of MRD. The results confirm the high sensitivity of HAT-PCR and show that its sensitivity is greater than that of flow cytometry. We conclude that, owing to its practical advantages and sensitivity, HAT-PCR merits being widely used for the measurement of MRD in lymphoid malignancy, particularly in acute lymphoblastic leukemia, to replace PCR as currently performed, and in myeloma.

## 2. Results

### 2.1. CLL

All 35 diagnostic samples corresponding to the samples used for MRD assays were sequenced by the Lymphotrack assay. For additional diagnosis samples, an IGH sequence was obtained by the Fr2:Fr3 protocol for 37 of 37 samples and a DJ sequence was obtained by the D protocol for 28 of 37 samples.

**Figure 1:**
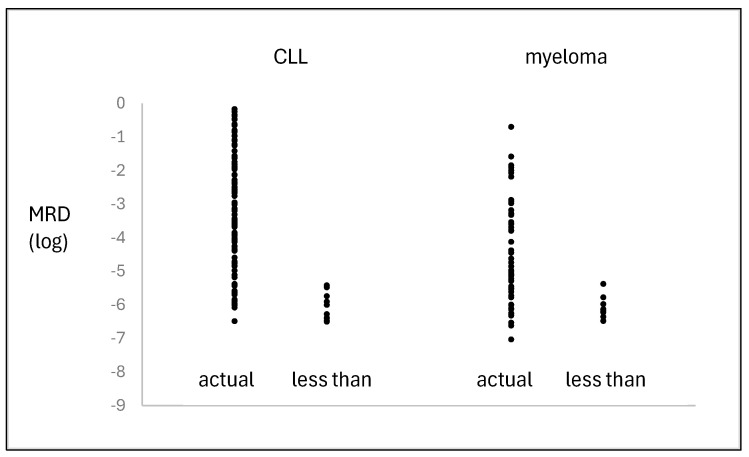
MRD results in chronic lymphocytic leukemia and myeloma assayed by HAT-PCR. The “less than” values refer to results in which MRD was not detected and, in view of the amount of DNA which was assayed, the true value was less than the value shown.

MRD was assayed in 125 samples, either blood (93) or marrow (32), which had been obtained from 36 patients at various times during treatment. The number of samples/patients ranged from one to nine, with a median of three. Non-specificity with HAT-PCR was only seen with primers from two patients, at levels equivalent to MRD values of 3.4 × 10^−7^ and 6.8 × 10^−7^. The MRD results measured by HAT-PCR are shown in Figure 1. There were seventeen MRD values between 10^−5^ and 10^−6^ and two measured values of less than 10^−6^.

**Figure 2:**
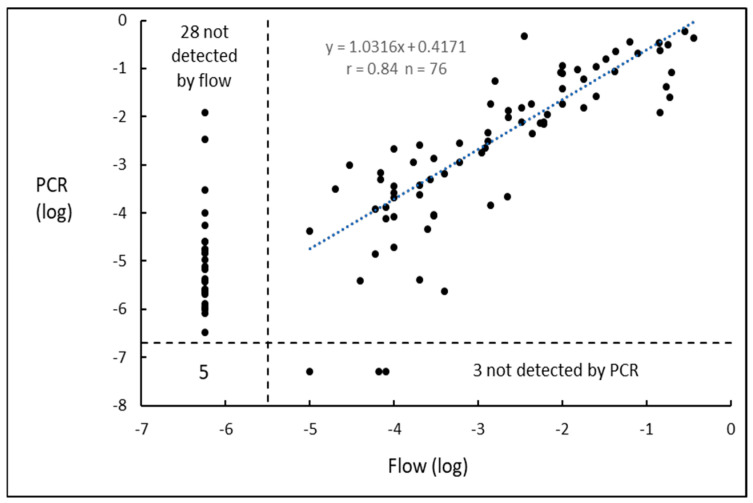
MRD results in chronic lymphocytic leukemia for samples assayed by both HAT-PCR and flow cytometry.

A total of 113 samples were analyzed by both flow cytometry and HAT-PCR. Flow measured down to 10^−5^ whereas HAT-PCR measured down to 3.4 × 10^−7^. The results are shown in Figure 2.

MRD was detected by both methods in 77 samples and there was excellent correlation (r = 0.84, *p* << 0.0001) between the methods. The level of MRD measured by HAT-PCR was slightly greater than the level measured by flow, with the ratio between HAT-PCR and flow being 1.98 (t = 3.31, *p* < 0.002). MRD was detected and quantified only by HAT-PCR in 28 samples and only by flow in 3 samples. This difference was highly significant (*p* < 0.001, binomial test) and indicated that HAT-PCR was more sensitive than flow. MRD was not detected by either modality in five samples.

Of the twenty-eight samples in which MRD was not detected by flow but was quantified by HAT-PCR, the level measured by HAT-PCR was between 10^−5^ and 10^−4^ in nine and was above 10^−4^ in three. These three samples had been transported interstate and two came from the same institution. Sample deterioration during lengthy transport may conceivably have played a role in the impairment of flow to detect MRD.

**Figure 3:**
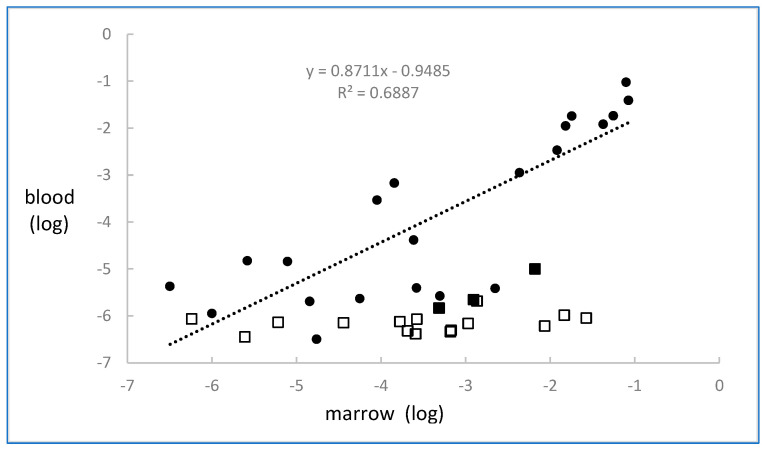
MRD results assayed by HAT-PCR in chronic lymphocytic leukemia and myeloma in paired samples of blood and marrow obtained on the same day. All samples had MRD detected and quantified in marrow. The closed circles are the MRD results for CLL blood samples, the closed squares are the MRD results for myeloma blood samples, and the open squares are the results for myeloma blood samples in which MRD was not detected, and they indicate that the level was less than the value shown. The regression line and equation are based on the CLL results.

There were 22 pairs of blood and marrow samples assayed by both flow and HAT-PCR. Results are shown in Figure 3. There was a highly significant correlation (r = 0.83, *p* < 0.00001) between the levels of MRD in blood and marrow. The mean level in marrow was 2.9 times that in blood (t = 2.13, *p* < 0.05).

### 2.2. Myeloma

Sequencing was performed in 35 patients. A sequence was obtained in 23/33 by the Fr1:Fr2 protocol, in 25/32 by the Fr1:Fr3 protocol, in 30/35 using the Fr2:Fr3 protocol, in 29/35 using the Fr3:Fr3 protocol, and in 14/33 using the D protocol. Overall, an IGH sequence only was obtained for 20 patients, a D sequence only was obtained for 2 patients, both IGH and D sequences were obtained for 12 patients, and no sequence was obtained for 1 patient.

MRD was quantified in 63 treatment marrow samples obtained from 23 of the 35 patients. The results are shown in Figure 1. A measured value was obtained in 51 samples and ranged from 2 × 10^−1^ to 2.4 × 10^−7^, with 10 samples being below 10^−6^. A measured value was not obtainable in 12 samples, with the results indicating that the MRD levels ranged from less than 4 × 10^−6^ to less than 3 × 10^−7^. False positivity was not seen in the control samples.

**Figure 4:**
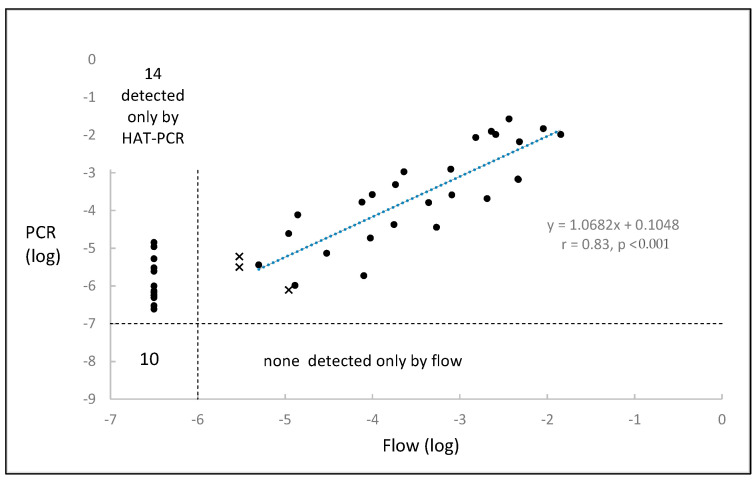
MRD results in myeloma for samples assayed by both HAT-PCR and flow cytometry. The “x” symbol refers to flow cytometry results, which were suspicious but not definite, owing to the detection of between 20 and 50 positive events. The suspicious results were not used for calculation of the regression equation.

MRD was measured by both HAT-PCR and flow in 51 of the 63 samples, and the results are shown in Figure 4.

The flow result was negative for 10 samples, scored as “suspicious” for 4 samples owing to between 20 and 50 positive events being recorded, and was a definite value for 37 samples. There were 37 samples which were quantifiable by both techniques with significant correlation being observed (r = 0.83, *p* < 0.001), 14 samples with MRD results in the 10^−5^–10^−7^ range which were quantifiable only by HAT-PCR, no samples which were quantifiable only by flow, and 10 samples which were not quantifiable by either technique. The difference between HAT-PCR and flow in the ability to detect and quantify was highly significant (*p* < 0.0001, binomial test).

MRD was also measured in 18 blood samples which had been obtained on the same date as the corresponding marrow sample. The results are shown in Figure 3 and, in contrast to the observations in CLL, blood MRD values were very low or not detectable when compared to marrow MRD levels.

## 3. Discussion

The generally accepted sensitivities [18,19] for the methods for the quantification of MRD are 10^−4^ to 10^−5^ for PCR, 10^−5^ for digital PCR, 10^−5^ for flow cytometry, and 10^−6^ for NGS. As seen in Figure 1, HAT-PCR was able to quantify down to and below 10^−6^, indicating that its sensitivity was superior to that of conventional PCR, digital PCR, and flow cytometry and was comparable to that of NGS. The greater sensitivity of HAT-PCR as compared to flow was shown in the direct comparison, which is illustrated for CLL in Figure 2 and for myeloma in Figure 4.

In addition to its sensitivity, HAT-PCR provides the advantages of PCR as it is simple, cheap, and immediately available if the institution has a molecular laboratory. Even if it does not, the sample is stable during transportation to an institution that does. The requirement for the determination of the DNA sequence of the rearranged Ig or TCR gene has been much simplified by the development of NGS. The disadvantages of HAT-PCR are that it does not directly detect clonal succession and that sensitivity may very occasionally be limited if the rearrangement has a short N region and a false-positive signal results.

The features of the other techniques used for the quantification of MRD may be compared to those of HAT-PCR.

Conventional PCR: This is 2 logs less sensitive than HAT-PCR and sometimes results in false-positive results in the 10^−4^–10^−5^ range. Its replacement by HAT-PCR would be technically trivial.

Digital PCR: This is slightly more sensitive than conventional PCR as the amplification of non-genomic sequences is partitioned separately from amplification of the target of interest, so that amplification of the target is not inhibited. However, false-positive results still occur. The design features of HAT-PCR could be combined with quantification by digital PCR, and dHAT-PCR could well have a sensitivity below 10^−5^.

Flow cytometry: This is widely used as it is readily available. It requires operator expertise, relatively fresh samples, and the procedure may be complicated by the therapeutic use of antibodies directed at key surface antigens.

NGS: This is the most sensitive technique, and it detects clonal succession. Two companies provide an NGS assay. The clonoSEQ method is provided by Adaptive Biotechnologies through selected reference laboratories. This method is expensive and requires transport to the reference laboratory, which leads to time delay and extra expenses. The Lymphotrack kit provided by Invivoscribe enables library preparation and provides bioinformatics but requires availability of an NGS facility.

Given these advantages and disadvantages, the potential role of HAT-PCR in the following diseases may be considered.

ALL: The Euro MRD consortium has promoted the use of conventional PCR for almost three decades and has published repeatedly on the methodology and the clinical results. The latest publication [4], in 2024, highlighted as outstanding issues the limited sensitivity of conventional PCR and the difficulty in distinguishing between false positives and true positives for results in the range of 10^−4^–10^−5^. HAT-PCR would almost entirely eliminate both issues as it minimizes promiscuous primer binding both to non-target IGH and TCR rearrangements and to non-target genomic sequences in general [17]. MRD levels between 10^−5^ and 3 × 10^−7^ would be measurable, and the accuracy of results in the 10^−4^–10^−5^ range would be increased.

Neither conventional PCR nor HAT-PCR deals directly with the problem of clonal succession. However, as the clones responsible are usually observed as minor clones identifiable by NGS at diagnosis [20], and as clonal succession is usually evident in the marrow at the end of induction, separate HAT-PCRs could be used to quantify the minor clones in the remission marrow and detect clonal succession. Less DNA would be used in each reaction, which would be slightly less sensitive, but given the overall sensitivity of HAT-PCR, the decrease in sensitivity would be minimal.

Flow cytometry is also widely used to quantify MRD in ALL. It is relatively cheap and widely available. Several recent publications have compared the use of flow cytometry and NGS and have suggested that NGS is superior, both in terms of sensitivity and of prediction of outcome [21,22,23]. As HAT-PCR has a sensitivity equivalent to that of NGS and is much cheaper and more readily available, it could be used as a substitute for flow. The issue of clonal succession could be handled as suggested above, or if multiple MRD assays are to be performed throughout a patient’s treatment, critical assays could be performed by NGS and less-critical assays by HAT-PCR.

CLL: Quantification of MRD by flow cytometry is widely performed. Clonal succession is uncommon in CLL but does occur. HAT-PCR would be more or less equivalent to flow cytometry in terms of cost and availability, it would be more sensitive, and the sample would be stable during transport, but it would need to be modified as suggested for ALL to deal with the issue of clonal succession.

Myeloma: Quantification of MRD is performed either by flow cytometry or by NGS. Myeloma is probably the ideal target for HAT-PCR owing to its sensitivity, the stability of the sample, the rarity of clonal succession, and the presence of hypermutation, which enables the design of semi-specific reverse J primers, thus further improving the specificity of the reaction. HAT-PCR seems to have real advantages over flow cytometry as it is more sensitive and the sample is stable. It has the advantages of cost, availability, and timeliness over NGS, and depending on further investigation, it may have an additional advantage as discussed below.

Figure 2 and Figure 4 show, for CLL and myeloma, respectively, the results for samples quantified by both HAT-PCR and flow. Four populations of samples are evident: (a) samples quantifiable by both methods, which show close to a 1:1 correspondence between the results of the two methods; (b) samples negative by both methods; (c) numerous samples that are flow-negative and HAT-PCR-positive. Such samples would be expected to occur owing to the greater sensitivity of HAT-PCR, and for a proportion of the CLL samples, to sample degradation, and (d) samples which are flow-positive and HAT-PCR-negative. Such samples were infrequent in CLL and not observed in myeloma. As HAT-PCR is more sensitive than flow, the infrequency or absence of samples that are flow-positive and HAT-PCR-negative is expected.

As NGS is more sensitive than flow, samples that are flow-positive and NGS-negative are similarly expected to be uncommon or absent. There are at least four reports for myeloma [24,25,26,27] and one for ALL [23], which provide visual data for individual samples quantified both by flow and NGS. Although interpretation of individual reports may be influenced by random variation and varying sensitivities of the methods used, all four myeloma reports show a population of flow-positive and NGS-negative results, and in several instances, a population of results which show NGS values disproportionately low as compared to the flow values. A similar population is uncommon in the ALL results and is completely absent in Figure 4 of the present report which shows results for samples quantified by flow and HAT-PCR. The likely explanation of the observation in myeloma of samples that are flow-positive and NGS-negative or low NGS is that the primers used for the preparation of NGS libraries are generic primers and the mutations in myeloma that occur in the rearranged IGH sequences interfere with primer binding and result in low or negative MRD values. This difference between HAT-PCR and NGS in assays of MRD needs investigation as it raises doubts concerning assays of MRD by NGS in myeloma and possibly in hypermutated CLL.

Our study also examined the relationship between levels of MRD in blood and marrow. In CLL, there was only an approximately three-fold difference between blood and marrow MRD levels, which indicates the possibility of using blood rather than marrow to quantify MRD in this disease. This could be performed either by flow or by HAT-PCR. In contrast to CLL, the blood MRD levels in myeloma were substantially lower than the levels in marrow. This phenomenon has been previously reported, but its existence has not posed a barrier to the potential use of blood for the monitoring of MRD in myeloma [28].

## 4. Materials and Methods

### 4.1. Samples

Samples of marrow and blood were obtained for measurement of MRD at various times during treatment from patients participating in studies CLL6 and MM17 of the Australasian Leukemia and Lymphoma Group. CLL6 studied patients diagnosed with B-CLL according to the WHO criteria and was a controlled study aiming to determine if lenalidomide consolidation was capable of extending progression-free survival. MM17 studied patients diagnosed with myeloma according to the International Myeloma Working Group diagnostic criteria and was a single-arm study of carfilzomib-thalidomide dexamethasone for newly diagnosed transplant-eligible patients refractory to initial bortezomib-based induction therapy.

The studies and the collection and storage of samples were approved by the relevant hospital ethics committees, all samples were obtained and stored after informed consent, and all patient details were anonymized. The samples were sent from various hospitals around Australia to two national collection points: the site of the flow cytometry laboratory in Sydney for CLL and the site of the flow cytometry laboratory in Melbourne for myeloma.

### 4.2. Quantification by Flow Cytometry

CLL: Samples were only processed if the time from collection was less than 72 h. Whole peripheral blood or bone marrow was lysed in bulk using ammonium chloride. Multiparameter flow cytometry [29] using 10 colours in a single tube was performed to assess MRD with a sensitivity of 10^−4^–10^−5^ by acquiring 2 million leucocytes with the limit of detection defined as 20 CLL cells. The method was validated against the international standardized flow cytometric approach for MRD detection in CLL (ERIC)-treated patients.

Myeloma: Samples were only processed if the time from collection was less than 48 h. The bulk lysing EuroFlow protocol provided a minimum of 5 × 10^6^ CD45 bone marrow cells for analysis. Samples were tested with a standardized EuroFlow™, 8 colour, two tube panel, and a minimum of 20 and 50 positive events were used to calculate the lower limits of detection and quantification, respectively, and the sensitivity was 10^−5^ for all bone marrow samples tested [24].

### 4.3. Quantification by HAT-PCR

#### 4.3.1. Determining the Sequence

For the CLL patients, the sequence of the IGH rearrangement was determined by NGS using the LymphoTrack kit (Invivoscribe, San Diego, CA, USA) according to the manufacturer’s instructions. The sequencing was originally performed to determine mutation status, but the sequence was also used to design primers.

For the myeloma patients, and a further cohort of CLL patients, the sequencing of the IGH rearrangement was also performed by NGS. To overcome the problem of hypermutation, the library for sequencing was prepared by a semi-nested PCR using high concentrations of forward primers for the Fr1, Fr2, and Fr3 regions of the IGH gene, multiple primers for each J region, prolonged annealing times, an annealing temperature of 20–25 °C below the Tm of the primers, and a polymerase with 3′–5′ exonuclease activity in the first round of PCR. Full details are given in Supplementary Data. The sequencing and bioinformatics analysis were performed at the Australian Genomic Research Facility.

#### 4.3.2. HAT-PCR

HAT-PCR is designed to provide maximum prevention of non-specific amplification. The important features are as follows:

1. One to four A/T bases at the 3′ end of both the allele-specific oligonucleotide (ASO) and J primers.
2. Annealing temperature within 3 °C of critical annealing temperature (Tc, the highest annealing temperature which still gives efficient amplification).
3. A relatively high concentration of Taq polymerase.

These features are designed to prevent non-specific amplification, which manifests either as a false-positive result or as failure of the PCR due to amplification of genomic sequences.

In practice, an annealing temperature of 72 °C was used for the PCR; the forward ASO primers were designed to have a Tm between 69 °C and 71 °C using the OligoAnalyzer tool from IDT (Coralville, IA, USA) and using the values of 5 mmol/L of Mg and 0.3 mmol/L of dNTPs, as these were the values used in the PCR. The reverse J primers were designed similarly except that their Tm was slightly higher, between 71.5 °C and 72.5 °C. An 8-well gradient of annealing temperature ranging from 68 to 75 °C was used to determine the Tc and confirm that the 72 °C annealing temperature fell between Tc and (Tc − 3).

The constituents of the PCR were as follows: KCl 50 mM, MgCl_2_ 5 mM, dNTPs 300 mM, primers 400 nM, probe 160 nM, Taq 4U, Tris HCl 20 mM (ph 8.4), H_2_O in a volume of 50 μL. The PCR amplification protocol was as follows: 91 °C for 3 min; 5 cycles of 97 °C for 15 s and 72 °C for 30 s; 5 cycles of 96 °C for 15 s and 72 °C for 30 s; 35 cycles of 94 °C for 15 s and annealing at 72 °C for 30 s.

Droplet digital PCR (dd-PCR) was performed using the Bio-Rad (Hercules, CA, USA) QX100 instrument according to the manufacturer’s instructions. Using dd-PCR, a reference gene, *GALT*, was used to assess DNA integrity of all samples, and for diagnostic samples, specific primers were used to determine the proportion of neoplastic cells, information which was necessary to construct the standard curve for the definitive assay.

In the definitive RT-qPCR assay for MRD, specificity for each primer was tested using at least 20 wells each containing 1 µg of DNA that had been extracted from blood samples from 5 non-leukemic individuals and then pooled. Quantification of patient samples was performed using 1 µg of DNA per well, with 3 wells being assayed if the Ct of a preliminary triage PCR was less than 35, otherwise 30 wells for samples from CLL, and depending on the availability of DNA, up to 20 wells for samples from myeloma patients. Quantification was performed and analyzed using a standard curve of dilutions of diagnostic DNA, but for samples with a low MRD level, it also proved possible to do a digital analysis based on the proportion of the assay wells which showed amplification. In these instances, we calculated the geometric mean of the analogue and digital values.

### 4.4. Statistical Analysis

All statistical tests were performed using the logarithms of the results of the MRD assays. The final estimates of the ratios between marrow and blood MRD levels are presented as arithmetic values.

Regression lines for the data illustrated in Figure 2, Figure 3 and Figure 4 were calculated using the LINEST function of Microsoft Excel, which is based on the assumption of normalized data and uses least-squares analysis.

The binomial test, as described on pages 36–42 of Siegel [30], was used to test the null hypothesis that the sensitivities of flow and HAT-PCR were identical. The hypothesis was tested separately for CLL and for myeloma, and the dataset for each comprised those results in which one method gave a positive result and the other gave a negative result and which are shown in the upper left and lower right quadrants of the respective Figure 2 or Figure 4. If the sensitivities of flow and HAT-PCR were identical, then the expected frequency of flow-positive and HAT-PCR-negative results would be the same as the expected frequency of flow-negative and HAT-PCR-positive results, with each expected frequency being 0.5. The results of the null hypothesis would resemble the results of a coin toss.

## Data Availability

The original contributions presented in this study are included in the article/Supplementary Materials. Further inquiries can be directed to the corresponding author.

## Supplementary Materials

The following supporting information can be downloaded at: https://www.mdpi.com/article/10.3390/ijms26167720/s1. One reference to D primers is included [31].
